# Stunting among Children Aged 6 to 59 Months Visiting the Outpatient Department of Pediatrics in a Tertiary Care Centre

**DOI:** 10.31729/jnma.8249

**Published:** 2023-08-31

**Authors:** Sharda Acharya, Bibechan Thapa, Rochak Kansakar, Henish Shakya, Ajaya Kumar Dhakal, Divya KC

**Affiliations:** 1 Department of Pediatrics, KIST Medical College and Teaching Hospital, Imadol, Lalitpur, Nepal; 2 Department of Surgery, Nepal National Hospital, Kalanki, Kathmandu, Nepal; 3 Department of Emergency Medicine and Intensive Care Unit, Kathmandu Valley Neuro and General Hospital, Bagdurbar, Kathmandu, Nepal

**Keywords:** *children*, *prevalence*, *stunting*, *undernutrition*

## Abstract

**Introduction:**

Stunting refers to low height for age, resulting from chronic undernutrition, often linked to poor socio-economic conditions, maternal health, infant care, and nutrition. It hinders children's physical and cognitive development. In Nepal, over half of children under five suffer from malnutrition. Despite efforts, stunting remains high but has decreased from 57% in 1996 to 25% in 2022. The aim of the study was to find out the prevalence of stunting among children aged 6 to 59 months visiting the outpatient Department of Pediatrics in a tertiary care centre.

**Methods:**

A descriptive cross-sectional study was conducted among children aged 6 to 59 months visiting the outpatient Department of Pediatrics in a tertiary care centre after obtaining ethical approval from the Institutional Review Committee from 27 April 2023 to 15 July 2023. Anthropometric measurements were taken. World health organization standard growth charts for Z score was used appropriately for the completed age in months and gender of the child. A pre-designed questionnaire was used for face-to-face interviews. Convenience sampling method was used. The point estimate was calculated at a 95% Confidence Interval.

**Results:**

Among 320 children, 46 (14.38%) (10.54-18.22, 95% Confidence Interval) children had stunting. Among those 46 children with stunting, 20 (43.47%) had severe stunting.

**Conclusions:**

The prevalence of stunting among children aged 6 to 59 months was found to be lower than other studies done in similar settings.

## INTRODUCTION

Stunting is the term used for low height for age, resulting from chronic undernutrition often linked to poor socio-economic conditions, maternal health, infant care, and nutrition. It hinders children from reaching their full physical and cognitive potential.^1^ It is a crucial indicator of child nutrition and health status.^2^

In Nepal, over half of the children under five years are estimated to suffer from some form of malnutrition.^3^ Despite efforts to improve child health and nutrition, stunting still remains high, although it has decreased from 57% in 1996 to 25% in 2022. Nepal government aims to reduce the prevalence of stunting among those under 5 years at or below 29% by 2022, and or below 15% by 2030 for sustainable development goal 2.1.1,^1^

The aim of the study was to find out the prevalence of stunting among children aged 6 to 59 months visiting the outpatient Department of Pediatrics in a tertiary care centre.

## METHODS

A descriptive cross-sectional study was conducted in the Outpatient Department of Pediatrics of KIST Medical College and Teaching Hospital, Imadol, Lalitpur, Nepal after receiving ethical approval from the Institutional Review Committee (Reference number: 2079/80/104). The study was done from 27 April 2023 to 15 July 2023. Children aged 6 to 59 months were enrolled in the study. Children with parents giving consent and presenting with acute illness or follow-up for acute illness were included. Children with congenital and chromosomal abnormalities, established chronic medical conditions (e.g., nephrotic syndrome, SLE), diagnosed endocrinological/hormonal diseases (e.g., diabetes, thyroid disorder, growth hormonal disorders), feeding difficulties (e.g., cleft lip/palate), digestion problems (e.g., celiac disease), neurodevelopmental disorders (e.g., cerebral palsy), long-term medication use (e.g., steroids), and active or treated long-term infections (e.g., TB, HIV) were excluded from the study. Convenience sampling was used. The sample size was calculated using the following formula:


$$\begin{array}{ll} \text{n} & ={\text{Z}}^{2}\times \frac{\text{p}\times \text{q}}{{\text{e}}^{\text{2}}}\\ & =1.96^{2}\times \frac{0.25\times 0.75}{0.05^{2}}\\ & =288 \end{array}$$

Where,

n = minimum required sample size
Z = 1.96 at 95% Confidence Interval (CI)
p = prevalence of stunting taken from previous studies, 25%^4^
q = 1-p
e = margin of error, 5%

The minimum required sample size was calculated to be 288. Adding 10% instrumental error, the sample size was 317. However, we took 320 samples.

A pre-designed questionnaire was formatted according to our objectives. A questionnaire was pretested among 30 children and necessary changes were made. The required information was taken from the parents or primary caretaker via face-to-face interviews. Anthropometric measurements were taken. The standing height of the child was measured with a stadiometer for children aged 2 years and older who are able to stand unassisted and recumbent length for the child was measured using an infantometer for those who could not stand.5 Reading was confirmed by two observers and the mean of two readings was taken.

World Health Organization (WHO) standard growth charts for Z score and percentiles^3^ were used appropriately for completed age in months and sex of the child. The Z score was calculated using the standard formula and plotted in a growth chart. Z score is below minus two standard deviations (-2 SD) from the median of the reference population are considered short for their age (stunted). Children who are below minus three standard deviations (-3 SD) are considered severely stunted.^4^

Data were entered and analyzed using IBM SPSS Statistics version 21.0. The point estimate was calculated at a 95% Confidence Interval.

## RESULTS

Among 320 children, 46 (14.38%) (10.54-18.22, 95% CI) children had stunting. Among those 46 children with stunting, 20 (43.47%) had severe stunting.

Out of all the stunted children, the majority were from the age group ≥12 to <24 months which was 18 (39.13%) with male preponderance with 27 (58.70%). All stunted children, 46 (100%) had access to safe toilet facilities and easy access to health care. Forty-five (97.83%) children had completed all immunization according to the extended immunization program of the Nepal Government (Table 1).

**Table 1:** Socio-demographic of children with stunting (n= 46).

Socio-demographic characteristics	n (%)
**Age (months)**	
≥6 to <12 months	4 (8.70)
≥12 to <24 months	18 (39.13)
≥24 to <36 months	8 (17.39)
≥36 to 48 months	8 (17.39)
≥48 to <60 months	8 (17.39)
**Gender**	
Male	27 (58.70)
Female	19 (41.30)
**Area of residence**	
Urban	42 (91.30)
Rural	4 (8.70)
**Ecological region of residence**	
Mountain	4 (8.70)
Hill	38 (82.60)
Terai	4 (8.70)
**Maternal education**	
Primary	4 (8.70)
Secondary	13 (28.26)
Higher secondary	19 (41.30)
Bachelors and above	10 (21.74)
**Maternal employment status**	
Employed	12 (26.09)
Not employed	34 (73.91)
**Type of family**	
Nuclear	31 (67.39)
Joint	15 (32.61)

**Figure 1:**
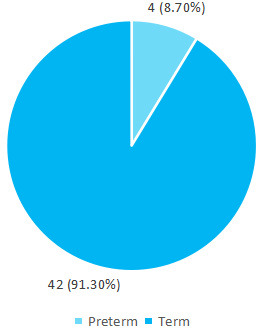
Gestational age at birth (n= 46).

Among 46 children, 44 (95.65%) were born at health institution and only 2 (4.35%) had delivery at home. The majority of the stunted children 42 (91.30%), were born at term (Figure 1).

The mean birth weight was 2879.78±512.59 grams whereas 6 (13.04%) children had low birth weight (birth weight <2500 grams) (Table 2).

**Table 2:** Birth history of the children with stunting (n= 46).

Parameters	n (%)
**Gestation status**	
Singleton	46 (100)
Multiple	0
**Low birth weight (birth weight <2500 grams)**	
Yes	6 (13.04)
No	40 (86.96)

The mean number of living children born before this child was 1.59±0.72. The majority, 24 (52.17%) had one living child born before this child (Table 3).

**Table 3:** Maternal obstetrical history of children with stunting (n= 46).

The number of living children born before	n (%)
this child	
One	24 (52.17)
Two	18 (39.13)
Three	3 (6.52)
Four	1 (2.17)
**Birth order**	
One	21 (45.65)
Two	21 (45.65)
Three	3 (6.52)
Four	1 (2.17)

Exclusive breastfeeding for 6 months was provided to 27(58.70%) of stunted children. The mean age of starting the complementary feeding was 5.80±1.44 months.

## DISCUSSION

Among 320 children visiting the outpatient Department of Pediatrics, 46 (14.38%) patients had stunting. In a previous study, it was found that the prevalence of stunting was found to be 25% which was slightly higher than in our setting.^5^ Similarly, in a study done in Nigeria, it was found that the prevalence of stunting was 36.7% which was higher than in our setting.^6^ The potential reason for a higher finding could be because the study was conducted at the national level which included children from low socio-economic status residing in rural areas as well whereas, in our study, most of the children 42 (91.30%) were from urban areas. A study showed that families residing in hill districts had less risk of stunting than those in the Terai plains.^7^ Our study had 38 (82.60%) residing in the hill districts and 4 (8.70%) residing in the Terai.

Studies have shown that babies born to uneducated women had a higher risk of stunting than those born to educated women.^7^ In our study, 4 (8.70%) of stunted children's mother had received primary education. Studies have shown that the prevalence of stunting was higher among children from larger families (38.7%)^2^ which was also seen in our study as 15 (32.61%) children were from joint families. For stunting, the common determinants identified were being from the highest equity quintile, being older, and being below the average size at the time of birth.^2^ In a study done in 1996, wealth (61%), caste/ ethnicity (12%), mother's education (12%), and birth order (9%) were the major contributors to observed socioeconomic inequalities in stunting; while in 2016, wealth (72%), mother's BMI (12%) and birth order (9%) were the major contributors.^8^

Inappropriate exclusive breastfeeding was found to be a risk factor for stunting in a previous study however in our study 27 (58.70%) of stunted children had received appropriate exclusive breastfeeding.^9^ In our study 6 (13.04%) children who were born low birth weight were stunted. A study suggests that low birth weight children are significantly more likely to be stunted as compared to children with normal birth weight.^10^ Likewise, a meta-analysis depicted that low birth weight could increase the incidence of stunting in children aged 0-60 months by 3.64 times to 6.95 compared to non-low birth weight children.^11^

Our study showed 4 (8.70%) children who were stunted had a preterm birth. Preterm birth is also considered a factor for stunting. Preterm birth has been demonstrated to be strongly related with child stunting in Indonesia.^12^ In a very large study, done in 137 developing countries, when multiple factors were considered, fetal growth retardation and preterm birth were the leading factors for stunting prevalence, with 32.5% of stunting prevalence being attributed to these factors.^13^

In our study, only 1 (2.17%) child who was stunted had not had immunization according to the extended national immunization schedule while a study done in Nepal found that children who had ever had vaccination were 62% less likely to be stunted than those who never had vaccination. It revealed that there is an increased risk of stunting if children are not vaccinated.^14^

The major limitation of this study is that this is single centered study so there is bias due to the catchment area. It is located in an urban area and therefore more people from urban areas with specific socio-demographic status may visit this centre. Also, this is a privately run hospital, therefore more people from a particular socio-economic class may visit this centre. Since this is a descriptive cross-sectional study conducted in a single setting, the results could not be generalized.

## CONCLUSIONS

In this study prevalence of stunting was lower than in other studies conducted in similar settings and national statistics. Regular use of anthropometric measurements in outpatient settings can effectively identify stunting. Early detection of this condition enables appropriate management, ultimately reducing potential complications.
